# Experimental proof that multivariate patterns among muscle attachments (entheses) can reflect repetitive muscle use

**DOI:** 10.1038/s41598-019-53021-8

**Published:** 2019-11-12

**Authors:** Fotios Alexandros Karakostis, Nathan Jeffery, Katerina Harvati

**Affiliations:** 1 Paleoanthropology, Senckenberg Centre for Human Evolution and Palaeoenvironment, Department of Geosciences, University of Tübingen, Tübingen, 72070 Germany; 2 Department of Musculoskeletal Biology, Institute of Ageing and Chronic Disease, University of Liverpool, Liverpool, L69 3GE United Kingdom; 3 DFG (Deutsche Forschungsgemeinschaft) Center for Advanced Studies “Words, Bones, Genes, Tools,” Eberhard Karls University of Tübingen, Ruemelinstrasse 23, D-72070 Tübingen, Germany

**Keywords:** Bone, Statistical methods, Image processing

## Abstract

Reconstructions of habitual activity in past populations and extinct human groups is a primary goal of paleoanthropological research. Muscle attachment scars (entheses) are widely considered as indicators of habitual activity and many attempts have been made to use them for this purpose. However, their interpretation remains equivocal due to methodological limitations and a paucity of empirical data supporting an interaction between systematic muscle forces and entheseal morphology. We have recently addressed the first issue with precise three-dimensional measuring protocols and rigorous multivariate analysis focusing on the patterns among different entheses rather than comparing each entheseal structure separately. In a previous study, the resulting entheseal correlations reflected synergistic muscle groups that separated individuals according to their lifelong occupational activities. Here we address the second issue by applying this methodology to existing micro-computed tomography data from rats that have undergone muscle stimulation under experimental conditions. In contrast to previous animal studies, we relied on blind analytical procedures across two research institutions and controlled for most factors of interindividual variability. Results demonstrated that the multivariate associations among different entheseal surfaces can directly reflect repetitive muscle recruitment and provide essential information on muscle use.

## Introduction

Entheses are the areas of the bone where muscles, tendons, or ligaments attach^1,2^. They represent the only direct evidence of the musculotendinous system on skeletal remains^2^ and are thought to broadly reflect muscular activity. As such, entheses have been used in attempts to reconstruct past habitual physical activity among past human populations and extinct hominin species^3–5^. Some of these studies^5–8^ rely on entheseal changes describing osteophytic or osteolytic traits within the broader area of muscle attachment, which can potentially be pathological^5,7,9^. By contrast, research focusing on variation in the entire entheseal morphology^10–19^ typically relies on the assumption that entheses vary in size and shape with changes of biomechanical load, according to Wolff’s law^20^ and to more recent paradigms of bone re-modeling before and during adulthood^21,22^. However, it has been demonstrated that the etiology underlying both aspects of entheseal variability (entheseal changes and entire enthesis morphology) is deeply multi-factorial, involving complex interactions among biological age, body size, sexual dimorphism, physical activity, population history, systemic factors (genes, nutrition and hormones), as well as pathology^4,5,9^. Detecting correlates of muscular activity through this maelstrom has been hindered in previous studies by important methodological design flaws, such as low or untested measuring precision^23^, lack of rigorous multivariate statistical approaches^16,24^, and disregard for important compounding variables such as old age and body size^4,9,25^. To address some of these issues, we recently presented a repeatable method for quantifying the three-dimensional (3D) surface areas of human hand entheses and multivariate analysis of the resulting measurements^15,16^. By focusing on the multivariate correlations among different entheses rather than on the morphology of individual entheses, we found that patterns within the human hand reflect two fundamental muscle synergy groups associated with power *versus* precision grasping movements^15^. We subsequently applied the method to a uniquely documented anthropological sample with known lifelong activities, exact work tasks, and life histories derived from the rare historical Basel Collection dated to the mid-19^th^ century. The results revealed a close association between multivariate patterns of entheseal morphology and the nature of lifelong activities^16^. All lifelong construction workers presented a power-grasping pattern, whereas long-term precision workers exclusively showed a precision grasping pattern involving the thumb and the index finger^16^. Importantly, these results were not associated with variations of age, body size, sex differences, socio-economic profiles, population of origin, pathological conditions, and inter-individual genetic variability^16,18^.

Nevertheless, some animal studies have argued against a functional character in entheseal morphology^11,14,19^, using diverse experimental conditions and species. Essentially, these works have concluded that habitual muscle recruitment does not have a significant effect on entheseal morphology. This has led to a growing skepticism among researchers as to the reliability of these structures for reconstructing past physical activity. However, these studies should not be considered definitive, as they present important methodological limitations and inconsistencies (see Materials and Methods; also see extensive discussion in Karakostis *et al*.^18^). Moreover, recent experimental research on adult mice entheses^26^ found that artificial muscle paralysis for 21 days led to significant bone loss in entheseal areas at the millimeter scale.

**Figure 1:**
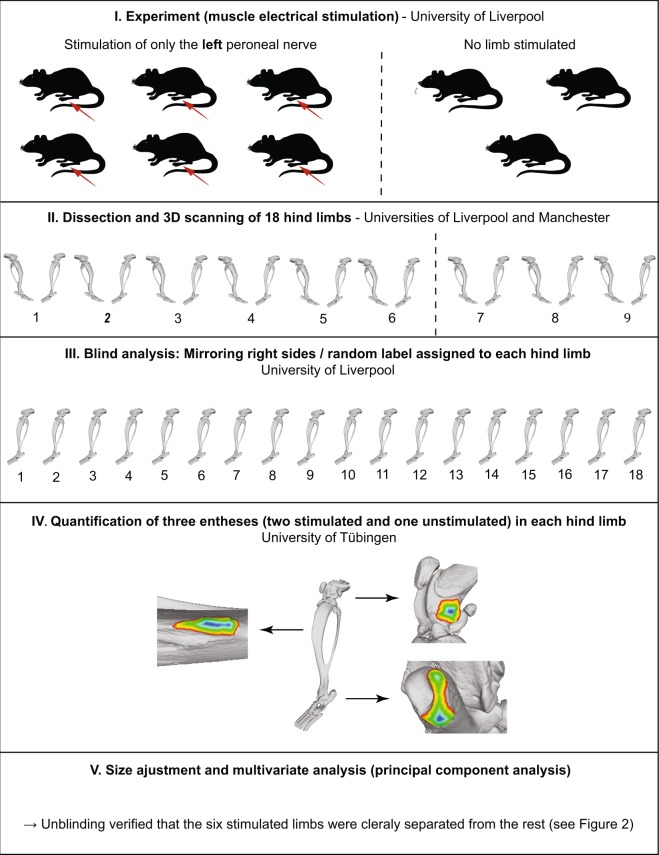
Steps of the fully blind experimental analysis, involving three different institutions.

The aim of the present study is to contribute to the current discussion by investigating the effects of increased muscle activation on entheseal multivariate patterns. We rely on a previously published animal model in which the common peroneal nerve was repeatedly stimulated in laboratory rats^27^. We address some of the shortcomings of previous animal studies by applying precise 3D measuring protocols and multivariate statistical analyses, focusing on the relationship among different entheses rather than each entheseal structure separately (see Karakostis *et al*.^16^). Our results show unprecedented experimental support that our recently developed multivariate 3D method^15,16,18^ can detect the effects of systematic muscle contraction on entheseal morphology under blinded study conditions (Fig. 1).

## Results

**Figure 2:**
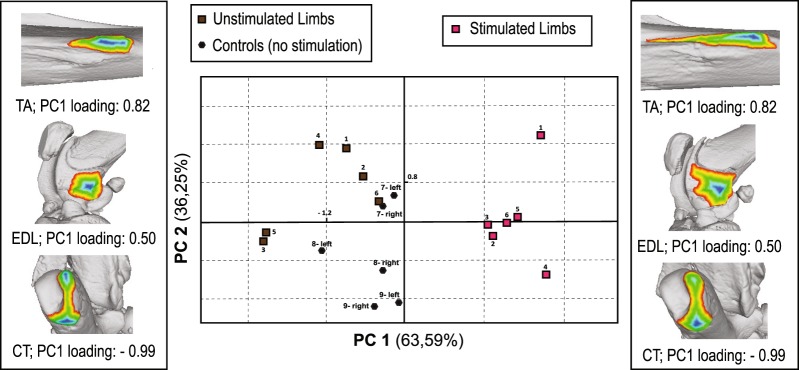
Results of the PCA on the size-adjusted entheseal 3D measurements of the 18 hind limbs from nine rat specimens. The analysis was fully blind and individuals were colored *a posteriori*. The side figures represent examples of morphological variation along PC1 between specimens with extreme negative (left) and positive (right) scores. Coloration is based on geodesic distances from the borders of entheses to their elevated center, and was performed using the tools of the open-access Meshlab software (CNR-INC, Rome, Italy).

Our analysis on size-adjusted 3D area measurements (Supplementary Table S1) provided a clear separation between two groups: one containing six datum points and the remaining 12 datum points forming a second group. Following decoding of the specimen IDs as per the blind study protocol (see Materials and Methods), these groups were found to correspond with the stimulated and unstimulated limbs, respectively. The results of the PCA, including plotted PC scores, factor loadings, and percentages of variation represented by each PC, are presented in Fig. 2 and Supplementary Table S2. Six of the 18 specimens (in purple) plotted far from the other 12 on PC1 (63.59% of total variation), presenting proportionally larger entheseal 3D areas of the two muscles contracted during the experiment (TA end EDL). The six unstimulated limbs of the same individuals (in brown) broadly overlapped with both limbs of the three control specimens (in black), showing proportionally larger CT, which is associated with muscles not stimulated in the experiment. These patterns were consistent with the extensive adaptations of muscle volume observed in the stimulated limbs (see Materials and Methods and Discussion).

As explained in Materials and Methods, additional steps were taken to further validate the robusticity of our results, in consideration of the relatively small sample size used in this controlled laboratory study. All three procedures support our main result (see PC1 in Fig. 2). Particularly, mean 95% confidence intervals for PC1 scores representing the six stimulated limbs were distinct from other limbs (1.31 to 2.09 compared with −1.31 to −0.39 points). This separation remained even after 1000 permutations (1.43 to 1.97 compared with −1.27 to −0.46 points). The t-test showed that the PC1 values of the stimulated hind limbs were significantly larger than the rest (p-value < 0.01; t-value = 8.05; mean difference = 2.55 points; standard error = 0.32 points) and remained so after 1000 permutations (p-value < 0.01, 95% confidence interval of the bootstrapped mean difference: 2.11 to 3.06 points). Unlike PC1, PC2 (36.25% of total variation) does not differentiate between stimulated and unstimulated limbs but scores were correlated with tibial length (p-value = 0.02, r-value = −0.56), which is a proxy for body size (see Table 1).

**Table 1:** Results of the ten correlation tests (statistically significant results are in bold).

Variables	Principal component 1		Principal component 2	
	p-value	r-value	p-value	r-value
Muscle volume	**<0.01**	**−0.61**	0.23	−0.30
Tibial length	0.82	0.06	**0.02**	**−0.54**
Anterior Cortical Thickness: Bone region 3 (proximal tibia)	0.35	0.24	0.56	0.15
Anterior Cortical Thickness: Bone region 5 (tibial midshaft)	0.24	−0.30	**0.02**	**−0.56**
Anterior Cortical Thickness: Bone region 9 (distal tibia)	**<0.01**	**0.76**	0.13	−0.37

The “bone regions” refer to 10 evenly distributed sampled sites along the surface of the tibia, spanning from the most anterior (number 1) to the most posterior (number 10) point of the bone.

A series of correlation tests were performed to evaluate the strength of association between each resulting entheseal pattern (PCs) and muscle volume (mm^3^) or average anterior cortical thickness (mm) in three different bone regions. The latter two variables were previously documented by Vickerton *et al*.^27^ (for more information, see Materials and Methods). The correlation tests revealed a strong link between PC1 scores and anterior cortical thickness (p-value < 0.01, r-value = 0.76) in a distal part of the tibia (i.e., region 9). In the previous study^27^, this particular bone region was associated with the highest levels of biomechanical stress during muscle contraction and increased new bone formation (also see Materials and Methods and Discussion). Correlations of PC1 scores with cortical thickness at regions of the midshaft and the proximal tibia were not significant (Table 1). By contrast, PC2 was only significantly correlated with anterior cortical thickness at the tibial midshaft (p-value = 0.02, r-value = −0.54; see Table 1), a measurement which did not show significant differences between stimulated and unstimulated limbs in the previous study by Vickerton *et al*.^27^. Importantly, unlike PC2, PC1 scores were strongly and negatively correlated with the volume of muscle *tibialis anterior* (p-value = 0.01, r-value = −0.61).

## Discussion

Our results demonstrate that repetitive muscle stimulation can be detected by analyzing multivariate patterns among different 3D entheseal surfaces. Particularly, our blind analytical procedures assigned high positive PC1 scores to all six stimulated limbs and negative values to all unstimulated contralateral limbs and controls. As predicted (for details, see Materials and Methods), the high PC1 values of stimulated limbs were associated with proportionally larger entheses of stimulated muscles TA and EDL, compared to CT (point of attachment of unstimulated muscles, including *gastrocnemius*). Our observations are generally consistent with the previous application of our method to human samples, which identified distinct entheseal patterns across individuals with well-documented differences of long-term habitual activity^16,18^. In contrast to that past research, however, the present study focuses solely on identifying the effects of frequent muscle stimulation on entheseal patterns and not the reconstruction of any particular pattern of long-term physical activity.

The factor loadings and PC1 scores reflected differences of muscle volume between stimulated and unstimulated limbs (Table 2; also see Materials and Methods). Directly stimulated muscles TA and EDL presented more than five times greater asymmetry than the unstimulated *gastrocnemius* (attaches onto CT). This pattern, which involved smaller muscle volume in the stimulated limbs, is a normal adaptation to reduce muscle fatigue by increasing the relative abundance of narrower fiber types^27^ (also see below). The results of a recent experimental study on adult mice established a direct association between unloading and bone loss at entheseal areas^26^. On this basis, the observed entheseal differences between stimulated and unstimulated limbs may be due to a combination of increased and decreased muscle use, respectively. It should be clarified that the high factor loading of CT on PC1 represents an effect of the size-adjustment based on the geometric mean (Supportive Table S1). Essentially, the relative proportion of CT surface area in these specimens was much larger compared to the stimulated individuals (which showed proportionally larger TA and EDL). In actual raw values (mm^2^), the mean difference in CT between stimulated and unstimulated limbs was small (5.53%).

**Table 2:** Means and standard deviations of muscle volume (mm^3^) in the left (stimulated) and the right (unstimulated) hind limbs of rats participating in the experiment as well as controls (no limb stimulated).

Group	Muscle	Left side	Right side	Mean relative difference (difference as % of right side)
Participating	TA	64.71 ± 10.95	80.34 ± 5.43	19.45%
	EDL	16.22 ± 2.05	19.52 ± 2.13	16.89%
	G	290.68 ± 15.70	299.82 ± 29.20	3.05%
Controls	TA	83.97 ± 8.58	86.74 ± 9.21	3.19%
	EDL	19.25 ± 2.11	19.79 ± 2.05	2.70%
	G	311.31 ± 28.97	310.67 ± 29.73	−0.21%

The muscles described are tibialis anterior (TA), extensor digitorum longus (EDL) and gastrocnemius (G).

While there is a clear clustering of stimulated limbs on PC1, PC2 likely reflects other factors of inter-individual variability (Fig. 2; Supplementary Table S2). This interpretation is supported by the very similar PC2 scores of the left and right sides of most individuals, and especially the control specimens (both limbs unstimulated). This is consistent with the significant correlation found between PC2 scores and tibial length (Table 1), which is directly correlated with body mass in Wistar rats^28^. Two of the stimulated rats (individuals 2 and 4), showed considerable bilateral asymmetry on PC2, suggesting that other, multi-etiological, factors contributed to entheseal variability. These observations are also broadly consistent with the results of our previous research on long-term physical activity in thoroughly documented humans, which showed that entheseal patterns were not identical among individuals with the same lifelong occupation and exact position at work^16^.

The multivariate nature of our analysis focuses on the proportions among different entheses within each individual rather than the morphology of each enthesis separately, striving to control for the multiple factors of inter-individual variation^1,2,4,9,16^. By contrast, the results of research focusing on the comparison of single entheseal structures across individuals or groups are often confounded by the effects of the numerous and complex factors driving interindividual variability in entheseal expression^4,5,8,9^. For instance, a recent study of entheseal changes^29^ compared the microscopic irregularity of human individual entheses with the cross-sectional robusticity of the corresponding bone. The authors reported insufficient bivariate correlations in a small documented sample with high age variability. However, when comparing the same structure across distinct individuals, the factors potentially affecting direct inter-individual variation in microscopic surface irregularity are truly numerous, spanning from genetic variability to cumulative pathology (e.g., micro-erosion, micro-porosis, and enthesophytes), nutrition, hormonal levels, muscle/tendon architecture, developmental conditions affecting bone modeling or remodeling, long-term physical activity patterns, or even taphonomy^1,2,4,9,16^.

The correlation tests (Table 1) found a strong association between PC1 scores and average anterior cortical thickness at an anterodistal point of the tibia. Particularly, among the 10 equidistant sampled sites of the tibia measured in Vickerton *et al*.^27^, this was region number 9. As outlined in Materials and Methods, the simulations of the finite element analysis performed on the same sample by Vickerton *et al*.^27^ suggested that this region of the tibia experiences high levels of strain during muscle stimulation. Interestingly, the region does not encompass the muscle attachment surfaces analyzed here and represented by PC1 (see Fig. 2). Therefore, it seems that the multivariate correlations among entheses reflect cortical thickness only at bone sites subjected to significant levels of stress and strain during muscle contraction, and not necessarily where each separate enthesis is located. This result suggests that future work on the correlation between entheseal surfaces and cross-section robusticity^14,19^ would greatly benefit from selecting the volume of interest on the basis of biomechanical predictions. This recommendation is further supported by the fact that anterior cortical thickness at midshaft was significantly correlated with the scores of PC2 (see Table 1), which were also associated with tibial length and did not show a clear functional signal (Fig. 2). Similarly, in Vickerton *et al*.^27^, cortical thickness at midshaft did not show significant differences between stimulated and unstimulated limbs (also see Materials and Methods).

The scores of PC1 also showed a strong negative correlation with the muscle volume of *tibialis anterior* (p-value = 0.01, r-value = −0.61). This outcome is also consistent with the results of Vickerton *et al*.^27^ based on the same sample (also see Materials and Methods). Particularly, in that previous study, the stimulated muscles showed a non-pathological adaptive decrease in volume coinciding with a transformation of the muscle fiber-type towards phenotypes which are less susceptible to fatigue (i.e., fiber types 2a and 1) but tend to present smaller cross-sectional areas and thus occupy a smaller muscle volume^27^. It is worth noting that similar muscle changes have been reported in studies of high endurance training both in rats and humans^30–32^. On this basis, the strong negative correlation reported here is important for understanding the mechanisms guiding entheseal variation in response to activity. It provides the first experimental indication that perhaps the most informative entheseal multivariate patterns only emerge when muscle recruitment becomes rigorous and differentiated enough to initiate muscle changes. Future experimental research could further explore the potential threshold required to initiate measurable entheseal variation by applying different loading regimes on different groups of laboratory species. A previous study on human cadavers has reported no bivariate correlation between two entheses of the hand and measurements of the attaching muscle (focusing on comparing each enthesis separately)^25^. The authors use this to argue against a functional character in entheses. However, the cadavers were from very old individuals (mean age 77.9 ± 12 years) and there are several well documented age-related effects that could have decoupled the relationship between muscle architecture and entheses, including diminished capacity for bone remodeling, lower levels of physical activity, as well as increased risk of sarcopenia and other comorbidities^5,9,16,18^. The potential effects of old age on the results of that previous work may be also aggravated by the lack of statistical control for the individuals’ body size.

Overall, our experimental analysis shows that the effects of repetitive muscle use on entheseal multivariate patterns can only overcome those of other variables when muscle contraction is rapid (high strain rate) and regularly repeated over relatively long periods of time. When this occurs, the signal of habitual stress in entheseal proportions becomes stronger than other sources of intraspecific variation. The effect of these other factors remain in the background (e.g. strong correlation between PC2 scores and tibial length; see Table 1) and can be adequately controlled (see Fig. 2) through size-adjusted and multivariate analyses of 3D entheseal areas precisely delineated using sophisticated imaging techniques^15–18^. Although the animal model used (developed in a previous study^27^) does not simulate specific real-life activity patterns (e.g., locomotion habits), our blind analysis of experimental data provided original proof that 3D entheseal multivariate patterns can directly reflect systematic contraction of specific muscles. This lends considerable technical credibility to our recently developed method of entheseal analysis, which has previously provided compelling evidence of an interaction between long-term occupational activity and entheses^16^. In the future, more nuanced cultural and behavior interpretations (e.g., subsistence strategies related to locomotion or manipulation) may come from the continued experimental application and development of the methodology described in the present study.

## Materials and Methods

### Participants and ethics statement

All *in-vivo* animal work carried out in the original study was approved by the Home Office (PPL 40/3280) and conducted in strict accordance with the Animals (Scientific Procedures) Act of 1986.

### Design of data collection and previous related experimental work

The present study is based upon data collected from a previous experimental study on Wistar rats^27^ designed to investigate the effects of electrical neuromuscular stimulation on bone morphology. We analyzed three entheses from the hind limbs of nine Wistar rats using 3D micro-computed tomography data. In six rats, an electrode was attached to the common peroneal nerve of the left hind limb with the right hind limb acting as the contralateral control. The remaining three individuals served as full controls. Stimulators delivered 0.2 ms pulses at 100 Hz for 200 ms every 30 s, resulting in a total of 9.6 minutes of stimulation per day for 28 days. The effective muscle contraction frequency experienced by the entheses was thus 0.03 Hz (approximately 2880 cycles per day). This effective frequency, which is actually lower than in many previous studies on rats reporting osteogenic responses^33,34^ (also see Qin *et al*.^35^), should not be confused with that of stimulating signal itself (100 Hz), which is used to recruit contraction of the muscle fibres and is not actually experienced by the bone during the stimulation process^33,34^.

**Figure 3:**
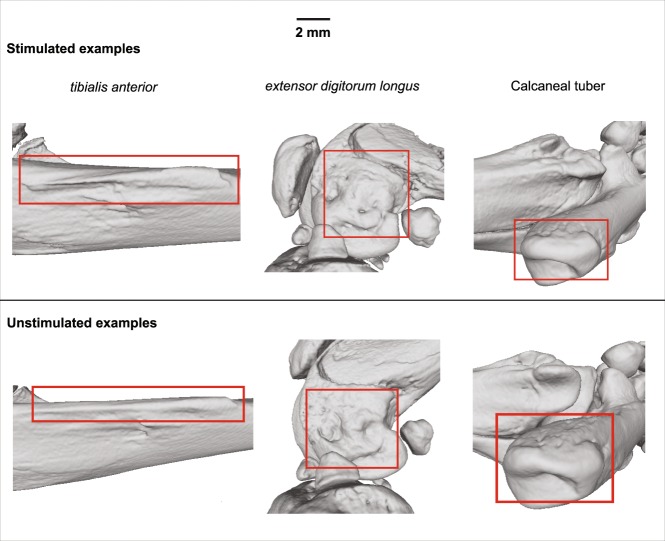
Examples of the three entheseal surfaces on the 3D models.

All rats were from the same population and the same living conditions, were sex- (males), strain- and age- (eight weeks) matched, and had similar body masses (228–282 g). Hind limbs were removed post-mortem, stored and then imaged with standard micro-computed tomography (microCT) and contrast-enhanced microCT using the Metris X-tek custom 320 kV bay system, (Manchester X-ray Imaging Facility) and reconstructed at 40 µm resolution (isometric voxels) (see Fig. 3 for examples of entheseal surfaces at this resolution). Studies of the gross morphology and histology showed no signs of pathology.

In the previous study, Vickerton *et al*.^27^ employed a combination of finite element analysis, morphological comparisons, and histological investigation to demonstrate the localized effects of repetitive muscle contraction on tibial bone volume (mm^3^), cross-sectional area (mm), and stress distribution. Cross-sectional measurements were represented by measurements of cortical thickness at 10 evenly distributed regions along the posterior and anterior tibial surfaces (where “region 1” comprises the most proximal sampled site and “region 10” represents the most distal one). Initially, the finite element analysis predicted that muscle stimulation would lead to substantial amounts of biomechanical stress on an anterodistal segment of the tibia. Focusing on that part of the bone (i.e., region 9), Vickerton *et al*.^27^ found that stimulated limbs present significantly greater anterior cortical thickness and bone volume than the unstimulated contralateral limbs of the same individuals, as well as both limbs of control specimens. The histological analysis of that region verified the presence of new bone formation encompassing clusters of chondrocytes. The remaining nine regions of the tibia did not present such evidence of a reaction to biomechanical stress. It is also worth noting that the stimulated muscles presented a significantly lower total volume (in mm^3^), coinciding with a transformation of muscle fibers towards types which are less susceptible to fatigue but present smaller cross-sectional areas^27^ (also see Discussion).

Applying the 3D entheseal analyses to the previously published rat model offers three distinct advantages compared with previous experimental works on entheses^11,14,19^. First, stimulating the muscles on one side provides a within-individual control, thus eliminating inter-individual variations of genes, hormones, nutrition, age, and body mass. If muscle recruitment affects entheseal patterns, the left stimulated limb is expected to present a different pattern of entheseal adaptation compared to the right limb in each rat. We also expect that, within each stimulated limb, the attachment areas of muscles innervated by the peroneal nerve would be proportionally larger than those of muscles not directly stimulated during the experiment. Moreover, if the effect is stronger than inter-individual variability, the six stimulated limbs are expected to differ to the twelve unstimulated limbs (six contralateral control limbs and six full control limbs). Second, contrary to previous works, the frequency and level of stimulation delivered were controlled and standardized for all test subjects. Finally, the measurements and analyses of the entheses were blinded. Each image file was randomly allocated a code by NJ before transfer from the University of Liverpool to FAK at the University of Tübingen. Also, image stacks for the right limb bones were mirrored so all limbs were presented to FAK as if they were from the left side of the body. Only after the analyses had been concluded by FAK were the datasets decoded to allow for differentiation between individuals and left and right hind limbs.

### Selection of entheses and predictions

It is widely known that the distinctiveness of entheseal areas on the bones varies greatly among and within individuals of the same species, with some muscle attachments being far more clearly defined on the bone than others^4,5,9,15^. Therefore, for the purpose of greater accuracy in our measurements, we selected three entheseal structures which were highly distinctive on the bone surface models (see Fig. 3) and directly homologous in all 18 lower limbs (Fig. 1). These were the insertion point of the calcaneal tuberosity (CT) as well as the two origin sites of *tibialis anterior* (TA) and *extensor digitorum longus* (EDL). Based on our own observations on the sample as well as published information on rat anatomy^36,37^, EDL originates from the lateral epicondyle of the femur, while TA arises from the anterior crest and lateral condyle of the tibia. For the latter muscle, we concentrated on the tibial crest because it was more clearly defined on the bones of our sample. In rats, TA and EDL are innervated by the common peroneal nerve and were thus repetitively contracted during the experiment, whereas the muscles associated with CT (i.e., the three major plantarflexors in rats, namely *soleus*, *gastrocnemius*, and *plantaris*) are innervated by the non-stimulated tibial nerve^36,37^.

It should be mentioned that the lateral epicondyle of the femur (enthesis of the stimulated EDL) also harbors an attachment site for *gastrocnemius*. Whilst there will be some indirect, antagonistic^36,37^, effect of the gastrocnemius, we do not expect this to be as marked as the influence of the directly stimulated muscles. This is fully supported by our observations on muscle volume data (Table 2; provided by the dataset used in Vickerton *et al*.^27^). These demonstrated that average volume differences were five times greater for directly stimulated muscles (TA and EDL) compared to the antagonistic *gastrocnemius* muscle (Table 2). We therefore predict that the proportional entheseal 3D size of the two stimulated muscles (TA and EDL) will differ to CT (attachment for muscles not directly stimulated, including *gastrocnemius*), leading the six stimulated left limbs to present distinctive multivariate patterns of entheses (e.g., see Karakostis *et al*.^16,18^).

### Multivariate 3D entheseal analysis

One of us (FAK) performed the extraction of the 3D surfaces from the 18 volume files and carried out the delimitation and quantification (in mm^2^) of the three entheseal surface areas (TA, EDL, and CT), following the highly repeatable method introduced in Karakostis *et al*.^15,16,18^. Briefly, this method relies on the combined use of several 3D imaging filters for delineating the exact borders of entheseal areas based on the criteria of bone elevation and surface complexity (i.e., calculation and color-mapping of the surrounding bone area’s principal directions of curvature, discrete curvature distribution, and geodesic distances within entheseal surfaces). Subsequently, the three obtained entheseal 3D measurements were size-adjusted for each hind limb separately using the geometric mean (Supplementary Table S1). The size-adjusted variables were then subjected to principal component analysis (PCA). This is a multivariate exploratory method that identifies patterns of variation within a sample without using *a priori* group classification. As reported above, FAK performed this analysis blind, so that the identities of the 18 limb bones were unknown. As the measurement scales of the three variables differed considerably, the PCA was based on the correlation matrix. The dataset met the assumptions required for PCA^38^, involving normal distribution (based on Shapiro-Wilk tests whose p-values ranged from 0.17 to 0.36), absence of outliers (based on the approaches involving z-scores as well as the interquartile range), minimum sample size requirements (five cases per variable), and factorability (based on a Bartlett’s test of sphericity showing a p-value of less than 0.01). Relying on the scree-plot technique^38^, we focused on the first two PCs (representing 99.84% of total variation). Following these analyses NJ decoded the specimen IDs.

Although the sample size was similar to those used in previous animal studies^19^, we undertook some additional statistics to explore the study power. First, we calculated the 95% confidence intervals for the mean of PC1 scores for each of the three groups (stimulated limbs, unstimulated contralateral limbs, and controls), in order to assess whether the values of stimulated limbs would theoretically maintain their distinctive patterns at a statistically assumed population level. Second, we performed bootstrapping (involving 1000 sample permutations) on the mean 95% confidence intervals of these PC1 scores^38^. Finally, we conducted an independent t-test for comparing the PC1 scores of the six stimulated limbs with the values of the 12 other limbs. The resulting statistics were also bootstrapped using 1000 permutations. Prior to the test, we verified that PC1 scores were normally distributed (Shapiro Wilk test’s p-value: 0.15) and contained no outliers^38^.

We then computed Pearson’s correlation coefficients, to test for the strength of association between each of the first two PCs and the volume of the muscle *tibialis anterior* (mm^3^), tibial anterior cortical thickness (mm), and tibial maximum length (measured directly on the 3D models in mm) as a proxy for individual body size^28^. Three measurements of anterior cortical thickness were used, representing three distinct regions of the tibia, relying on the data published by Vickerton *et al*.^27^ (for more information on the regions, see in the above subsection of Materials and Methods). These corresponded to the proximal area of the bone bearing part of the tibial crest (“region 3” in Vickerton *et al*.), the bone midshaft (“region 5”), and a distal part of the tibia (“region 9”), which showed a consistent functional signal in Vickerton *et al*.^27^. The results for the comparisons performed (p-values and r-values) are reported in Table 1.

## Supplementary information

Supplementary Information

## Data Availability

The numerical data presented in the current study are available from the corresponding author on reasonable request.
