# Supplementary material for: Experimental proof that multivariate patterns among muscle attachments (entheses) can reflect repetitive muscle use
Source: Sci Rep. 2019 Nov 12;9:16577. doi: 10.1038/s41598-019-53021-8 (PMC6851080; doi:10.1038/s41598-019-53021-8)
Supplement: Supplementary file 1 — Supplementary Information [file 41598_2019_53021_MOESM1_ESM.pdf]

Title: Experimental proof that multivariate patterns among muscle attachments (entheses) can reflect repetitive muscle use

Authors: Fotios Alexandros Karakostis[1*], Nathan Jeffery[2], Katerina Harvati[1,3]

Affiliations:

[1]Paleoanthropology, Senckenberg Centre for Human Evolution and Palaeoenvironment, Department of Geosciences, University of Tübingen, Tübingen 72070, Germany.

[2] Department of Musculoskeletal Biology, Institute of Ageing and Chronic Disease, University of Liverpool, Liverpool, L69 3GE, United Kingdom

[3]DFG (Deutsche Forschungsgemeinschaft) Center for Advanced Studies "Words, Bones, Genes, Tools," Eberhard Karls University of Tübingen, Ruemelinstrasse 23, D-72070 Tübingen, Germany.

*Corresponding author; E-mail: fotios-alexandros.karakostis@uni-tuebingen.de

Supplementary Table S1. Descriptive statistics for size-adjusted entheseal 3D measurements

**Entheses**	**Stimulated limbs**				**Non-stimulated limbs**				**Control group**			
	**Range**	**Mean**		**Standard Deviation**	**Range**	**Mean**		**Standard Deviation**	**Range**	**Mean**		**Standard Deviation**
		**Statistic**	**Standard Error**			**Statistic**	**Standard Error**			**Statistic**	**Standard Error**	
Tibialis anterior	0.13	0.72	0.02	0.04	0.09	0.56	0.01	0.03	0.11	0.65	0.02	0.05
Extensor digitorum longus	0.35	1.74	0.05	0.12	0.30	1.66	0.05	0.13	0.30	1.53	0.05	0.13
**Calcaneal tuber**	0.08	0.80	0.01	0.03	0.20	1.09	0.03	0.09	0.12	1.01	0.02	0.04

Supplementary Table S2. Statistics of the size-adjusted principal component analysis

**Principal component**	**Eigenvalue**	**% of variance**	**Factor loadings**		
			Tibialis anterior	***Extensor digitorum longus***	***Calcaneal tuber***
**1**	1.91	63.59	0.82	0.50	-0.99
**2**	1.09	36.25	-0.58	0.87	-0.04
**Total**		99.84			
